# HOCl can appear in the mitochondria of macrophages during bacterial infection as revealed by a sensitive mitochondrial-targeting fluorescent probe†
†Electronic supplementary information (ESI) available: Experimental section and supporting figures. See DOI: 10.1039/c5sc01562f



**DOI:** 10.1039/c5sc01562f

**Published:** 2015-06-01

**Authors:** Jin Zhou, Lihong Li, Wen Shi, Xinghui Gao, Xiaohua Li, Huimin Ma

**Affiliations:** a Beijing National Laboratory for Molecular Sciences , Key Laboratory of Analytical Chemistry for Living Biosystems , Institute of Chemistry , Chinese Academy of Sciences , Beijing 100190 , China . Email: shiwen@iccas.ac.cn ; Email: mahm@iccas.ac.cn

## Abstract

Macrophages, important cells of the innate immune system, can produce abundant HOCl in the cytoplasm to fight against bacteria. Recent studies suggest that mitochondria in macrophages play a role in antibacterial responses. During bacterial infection, however, it is uncertain whether HOCl is present in the mitochondria, mainly because of the lack of a suitable research method. Herein, by developing a new mitochondrial-targeting fluorescent HOCl probe, combined with confocal fluorescence imaging, we show for the first time that HOCl can appear in the mitochondria of macrophages (Raw264.7 cells) during bacterial infection, as confirmed with non-phagocytic cells and inhibitors as control experiments. Moreover, the developed probe exhibits an accurate mitochondrial-targeting ability, a fast response, and high selectivity and sensitivity (detection limit 9 nM), and is thus expected to be employed for further revealing the biological function of subcellular mitochondria.

## Abstract 2

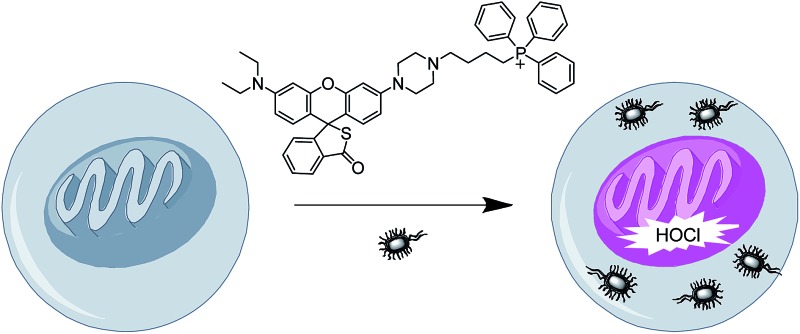
HOCl can appear in the mitochondria of macrophages during bacterial infection as revealed by a new sensitive mitochondrial-targeting fluorescent probe.

## Introduction

Macrophages are important cells of the innate immune system that are formed in response to an infection or an accumulation of damaged or dead cells. They can produce abundant HOCl in the cytoplasm to kill bacteria.^1,2^ The generation of the cytoplasmic HOCl is known to be mainly from phagosomes *via* the reaction of H_2_O_2_ and chloride ions in the presence of myeloperoxidase.^1^ However, current evidence has suggested that mitochondria in macrophages also play a role in the innate immunity,^3^ although it is unclear whether HOCl is present in the mitochondria. Interestingly, mitochondria are recognized as a significant source of reactive oxygen species (ROS), including H_2_O_2_, in most cells,^3–5^ and very recently myeloperoxidase has been found to be present in the mitochondria of macrophages.^6^ Thus, we can make a reasonable inference that mitochondria may also contribute to the total cellular HOCl during bacterial infection; however this has not yet been confirmed, primarily because of the lack of a suitable research method. Herein, by developing a new sensitive mitochondrial-targeting fluorescent HOCl probe, combined with confocal fluorescence imaging, we demonstrate that HOCl can indeed appear in the mitochondria of macrophages (Raw264.7 cells) during bacterial infection, possibly due to the mitochondria themselves generating HOCl. Furthermore, this observation is also confirmed by different control experiments such as *N*-acetylcysteine (NAC; a scavenger of HOCl), 4-aminobenzoic acid hydrazide (a specific inhibitor of myeloperoxidase), and nonphagocytic cells. Below we report these results.

The HOCl assay has attracted much attention due to its pivotal antimicrobial nature,^1^ and in this respect fluorescent probes^7–16^ have been widely used because of their high sensitivity and unrivaled spatiotemporal resolution.^17–19^ To explore whether HOCl can appear in the mitochondria of macrophages infected by bacteria, such as *Escherichia coli* (*E. coli*), the fluorescent HOCl probe needed should meet the following requirements: (a) mitochondrial-targeting ability, (b) high sensitivity (detection limit < 10 nM) and superior selectivity to accurately monitor HOCl generation, and (c) relatively long analytical wavelength (>550 nm) to minimize autofluorescence and biological damage. Unfortunately, a fluorescent probe simultaneously possessing these desired properties has been hitherto unavailable.

**Scheme 1:**
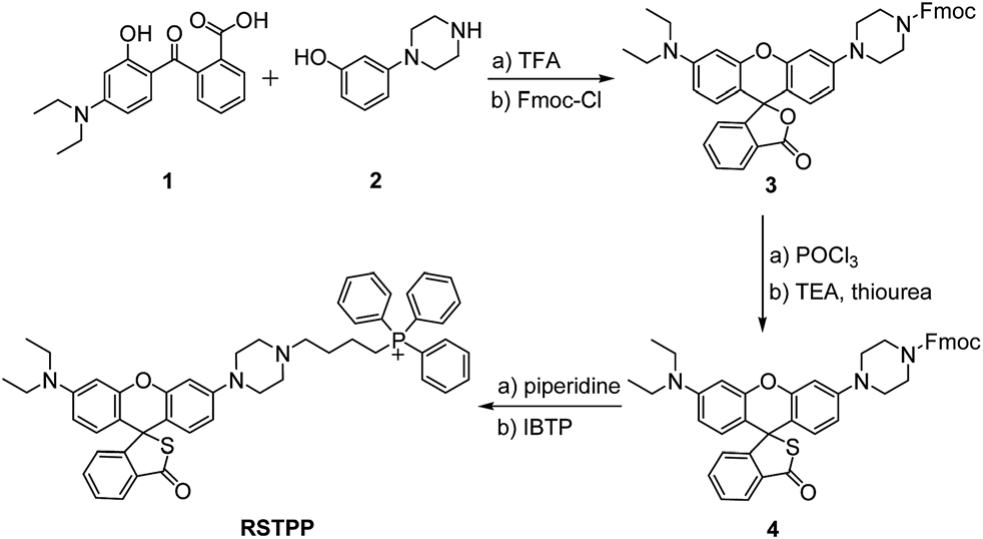
Synthesis of RSTPP.

In this work, we have developed such a HOCl probe, the rhodamine thiolactone triphenylphosphonium cation (RSTPP; Scheme 1), by engineering a typical mitochondrial-targeting moiety of the triphenylphosphonium cation^20^ into a crucial spectroscopic and recognition moiety of rhodamine thiolactone. We chose rhodamine thiolactone on the basis of the following two facts: first, we had previously reported on rhodamine B thiolactone, and extensive research reveals that it only reacts with Hg^2+^ and OCl^–^.^21–23^ Because the concentration of Hg^2+^ in biosystems is negligible, we envisioned that the rhodamine thiolactone skeleton may serve as a specific recognition unit for HOCl. Second, the structural change between the spirocyclic and spiroring-opening forms of rhodamine has been proven to be an efficient way to synthesize spectroscopic off–on probes for different analytes,^7^ and, in particular, the introduction of a sulfur atom, with its strong electron-donating ability,^24^ into the spirocyclic structure would further favor the fluorescence quenching of rhodamine. We conceived that this could make the resulting probe possess a lower fluorescence background signal, thereby achieving a higher detection sensitivity.

RSTPP can be prepared by first synthesizing a rhodamine intermediate that bears a protected piperazine handle, then incorporating the S atom into the spirocyclic structure, and finally linking the mitochondrial-targeting moiety of the triphenylphosphonium cation to the skeleton of the rhodamine thiolactone *via* the piperazine handle (Scheme 1). The obtained probe was well characterized by NMR and mass spectral analyses (Fig. S1–S4 in the ESI†), in which the typical quaternary carbon on the spiroring appears at 62 ppm in the ^13^C NMR spectrum, proving the formation of the thiolactone.

## Results and discussion

### Spectroscopic response of RSTPP to HOCl

**Fig. 1:**
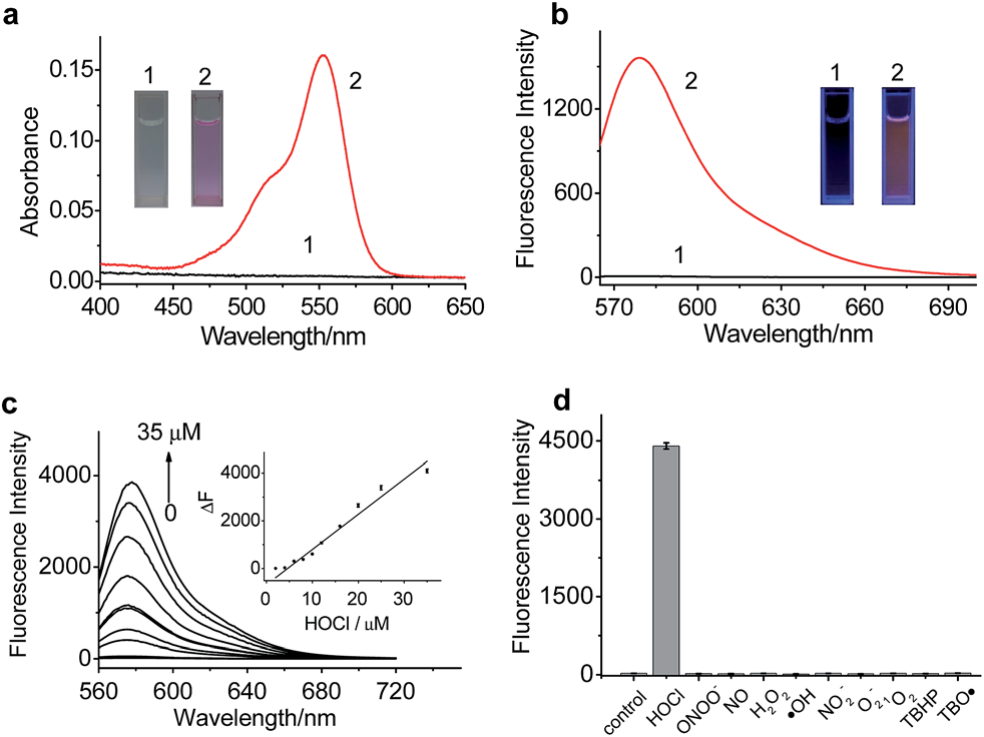
Spectroscopic properties of RSTPP. (a) Absorption and (b) fluorescence emission spectra of RSTPP (2.5 μM) in pH 7.4 PBS (1) before and (2) after reaction with HOCl (2.5 μM) for 2 min. Insets: the corresponding color changes of RSTPP before and after the reaction. (c) Fluorescence response of RSTPP (10 μM) to HOCl at varied concentrations (0–35 μM). Insert: linear fitting curve of Δ*F* against the concentration of HOCl. Δ*F* is the fluorescence intensity difference of RSTPP with and without HOCl. (d) Fluorescence responses of RSTPP (10 μM) to various ROS in PBS (pH 7.4): HOCl (100 μM), ONOO^–^ (200 μM), NO (100 μM), H_2_O_2_ (100 μM), ˙OH (100 μM), NO_2_^–^ (100 μM), O_2_^–^ (100 μM), ^1^O_2_ (100 μM), TBHP (100 μM), and TBO˙ (100 μM). *λ*_ex/em_ = 553/580 nm.

The spectroscopic properties of RSTPP are shown in Fig. 1. As expected, the probe itself is nearly colorless and nonfluorescent (Fig. 1a and b), and the extremely low background signal is rather favorable to sensitive detection. Upon the addition of HOCl, however, a big absorption peak at 553 nm and a large fluorescence emission at 580 nm appear, accompanied by a distinct color change from colorless to pink (insets of Fig. 1a and b). The fluorescence quantum yield of RSTPP is below 0.01, but rises to 0.34 in the presence of HOCl. This large contrast leads to a more than 200-fold increase in the fluorescence intensity. The enhanced fluorescence is indicative of the oxidative cleavage of the thiolactone ring triggered by HOCl, followed by desulfurization (–SCl) and the conjugated rhodamine formation, which was verified by mass spectral analysis (*m*/*z* = 772.3669 [M]^+^; Fig. S5 in the ESI†).

The reaction conditions for RSTPP with HOCl were optimized (Fig. S6†). The probe hardly emits fluorescence in a wide pH range of 3.0–9.0, indicating the insensitivity of the thiolactone form to the environmental pH change. The reaction of RSTPP with HOCl produces an almost invariant fluorescence in the pH range of 6.5–8.5 (Fig. S6a; ESI†), which covers the physiological pH range of mitochondria well (about pH 7.99).^25^ Notably, over 90% of the fluorescence reaction of the probe with HOCl is completed within 1 min and the maximum fluorescence remains unchanged for at least 1 h, whereas RSTPP itself scarcely shows any background fluorescence (Fig. S6b; ESI†). This high stability of RSTPP, together with its fast response, is rather important for the real-time sensing of HOCl in organisms. In the present work, a reaction time of 2 min was employed to achieve high reproducibility and accurate measurements.

Under the above-determined conditions, the fluorescence of RSTPP exhibits a good linear response to HOCl in the concentration range of 2.0 to 35 μM, with a regression equation of Δ*F* = 140.2 × [HOCl] (μM) – 499.9 (Fig. 1c). The detection limit (3*S*/*m*, where *S* is the standard deviation of 11 blank measurements, and *m* is the slope of the linear equation) was determined to be as low as 9 nM, which makes the probe feasible for monitoring the generation of mitochondrial HOCl at trace levels. Obviously, the high sensitivity of the probe is due to the combined usage of the strong electron-donating S atom and the spirocyclic structure of rhodamine.

Next, we studied the specificity of the probe for HOCl over other ROS (Fig. 1d), demonstrating that, except for HOCl, all the other ROS tested do not trigger the noticeable fluorescence enhancement. Moreover, the fluorescence responses of RSTPP to other biologically relevant species, such as amino acids, glutathione, human serum albumin, glucose, and inorganic salts, were examined, and no obvious change in fluorescence signal was detected in the presence of these species at their considerable concentrations when compared to the control (Fig. S7; ESI†). This indicates that RSTPP shows a high selectivity for HOCl over various potential interfering substances. In addition, RSTPP displays good biocompatibility (Fig. S8 in the ESI†), which makes it promising as a fluorescent probe for the selective and sensitive measurement of HOCl in biosystems.

### Mitochondrial-targeting properties of RSTPP in living cells

**Fig. 2:**
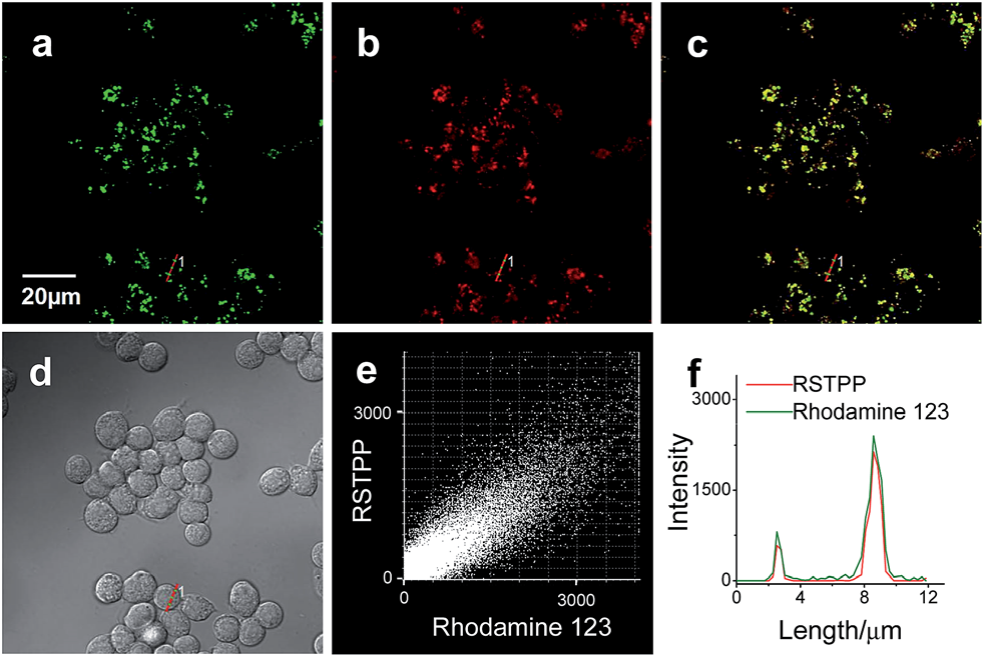
Colocalization of rhodamine 123 and RSTPP in macrophages (Raw264.7 cells). Cells were co-stained with rhodamine 123 (500 nM) and RSTPP (10 μM), and then treated with HOCl (50 μM) at 37 °C for 20 min. (a) Fluorescence image from the rhodamine 123 channel (*λ*_ex_ = 488 nm, *λ*_em_ = 495–550 nm). (b) Fluorescence image from the RSTPP channel (*λ*_ex_ = 559 nm, *λ*_em_ = 570–670 nm). (c) Merged image of images (a) and (b). (d) Corresponding differential interference contrast (DIC) image. (e) Intensity correlation plot of rhodamine 123 and RSTPP. (f) Intensity profiles of rhodamine 123 and RSTPP within the linear ROI 1 (red lines in (a) and (b)) across the Raw264.7 cell.

To examine the mitochondrial-targeting performance of RSTPP, co-localization experiments were conducted by co-staining macrophages with rhodamine 123 (a typical mitochondrial tracker) and RSTPP. The fluorescence of rhodamine 123 (Fig. 2a, green) from the co-stained cells in the presence of HOCl overlaps well with that of RSTPP (Fig. 2b, red), as shown in the merged image (Fig. 2c). Moreover, a high Pearson's coefficient of 0.92 and an overlap coefficient of 0.91 are obtained from the intensity correlation plots (Fig. 2e). Notably, the changes in the intensity profiles of the linear region of interest (ROI) 1 across the cell are synchronous in the two channels (Fig. 2f). Similar results were obtained for HeLa cells (Fig. S9 in the ESI†). This indicates that RSTPP can specifically target the mitochondria of living cells with good cell-membrane permeability. On the other hand, a negative control experiment was performed by co-staining Raw264.7 cells with Lyso Tracker Green DND-26 (DND-26, a lysosome-targeting dye) and RSTPP. In the presence of HOCl, the co-stained cells exhibit significantly different fluorescence regions for both DND-26 and RSTPP, accompanied by a rather poor Pearson's coefficient of 0.30 and an overlap coefficient of 0.28; furthermore, completely different changes in the intensity profiles of the linear ROI 1 were found (Fig. S10; ESI†). Also, similar phenomena were observed for HeLa cells (Fig. S11 in the ESI†). These findings further confirm the accurate mitochondrial-targeting ability of RSTPP in living cells.

### Fluorescence imaging of endogenous HOCl in living cells

Having demonstrated the mitochondrial-targeting ability of RSTPP, the probe was then preliminarily studied to detect the formation of endogenous mitochondrial HOCl in a known model; that is, whether macrophages, such as RAW264.7 cells, under the stimulation of lipopolysaccharide (LPS) and phorbol 12-myristate 13-acetate (PMA) could produce endogenous HOCl.^10,13^ The results (Fig. S12 in the ESI†) showed that the stimulated RAW264.7 cells displayed strong fluorescence; moreover, the fluorescence enhancement can be largely inhibited by NAC (a scavenger of HOCl). This demonstrates that RSTPP is capable of monitoring the generation of endogenous HOCl in the mitochondria of living macrophage cells. Another control experiment was carried out by treating RSTPP-loaded HeLa cells with NAC and then with HOCl, and a similar fluorescence-inhibiting effect by NAC was observed (Fig. S13 in the ESI†), further supporting the idea that the fluorescence of the probe-loaded cells arises from the action of HOCl.

### Detection of mitochondrial HOCl in macrophages infected by bacteria

**Fig. 3:**
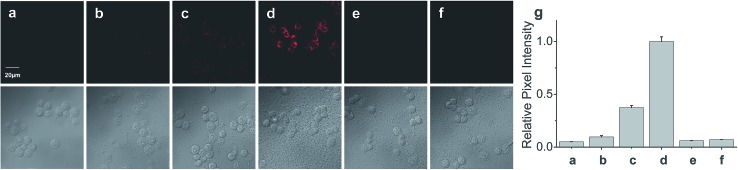
Fluorescence images of RAW264.7 cells during *E. coli* infection. (a) Cells were incubated with 10 μM RSTPP for 20 min (control). Cells were pretreated with *E. coli* at a concentration of 5 × 10^6^ CFU mL^–1^ for (b) 1 h, (c) 3 h, and (d) 7 h, and then incubated with 10 μM RSTPP for 20 min. (e) Cells were pretreated with 5 × 10^6^ CFU mL^–1^ *E. coli* for 7 h, then incubated with 10 μM NAC for 10 min, and finally incubated with 10 μM RSTPP for 20 min. (f) Cells were pretreated with 10 μM of 4-aminobenzoic acid hydrazide (a specific inhibitor of myeloperoxidase) for 10 min, then infected with 5 × 10^6^ CFU mL^–1^ *E. coli* for 7 h, and finally incubated with 10 μM RSTPP for 20 min. The corresponding DIC images of the fluorescence images are shown below them. (g) Relative pixel intensity measurements (*n* = 3) from images (a)–(f) by the software ImageJ. The pixel intensity from image (d) is defined as 1.0.

Finally, the probe was used to explore whether HOCl can appear in the mitochondria of macrophages during bacterial infection, with an experiment in which RAW264.7 cells as a model were infected by *E. coli* for different periods of time and RSTPP was employed to monitor the fluorescence change in real time *via* confocal fluorescence imaging (Fig. 3). It is found that RAW264.7 cells infected with *E. coli* produce significant fluorescence in the mitochondria, and the fluorescence intensity increases gradually over time (Fig. 3b–d). Interestingly, a rather proportional increase in the fluorescence intensity is observed with the infection time (Fig. 3g), and further infection leads to the culture media turning slightly yellow (data not shown), suggesting that the nutrients are no longer sufficient for the bacteria and cells. Moreover, an effective inhibition of the mitochondrial fluorescence by NAC was observed (Fig. 3e), proving that the fluorescence enhancement in the mitochondria during the *E. coli* infection is indeed due to the appearance of HOCl. The experiment with 4-aminobenzoic acid hydrazide, a specific inhibitor of myeloperoxidase,^26^ showed that the introduction of the inhibitor into the cells markedly decreases the mitochondrial fluorescence (Fig. 3f; compared to Fig. 3d). This can be ascribed to the strong suppression of the myeloperoxidase activity by the inhibitor, thereby decreasing the HOCl content. On the other hand, a comparative study was made under the same bacterial infection conditions using HeLa cells as a negative control, because HeLa cells, unlike phagocytes, are known to express very low levels of myeloperoxidase.^27^ The results showed that, after infection with *E. coli*, the HeLa cells cannot give any obvious mitochondrial fluorescence as RAW264.7 cells do (Fig. S14†), which further verifies that the mitochondrial fluorescence in RAW264.7 cells is attributed to the appearance of HOCl. Besides, it is noted that the fluorescence intensity from RAW264.7 cells is about 19 times higher than that from HeLa cells (Fig. S14e†). Supposing that the reaction properties of the probe in the two cell lines are equal, the concentration of HOCl generated in the mitochondria of RAW264.7 cells would be 19 times larger than that in HeLa cells, which provides the first semi-quantitative information about the HOCl contents in these two cell lines during bacterial infection. Our findings reveal that mitochondria, not just phagosomes, may also produce HOCl in the case of bacterial infection, though the possible diffusion of the cytoplasmic HOCl into mitochondria cannot be ruled out.

## Conclusions

In conclusion, we have developed a new fluorescent HOCl probe, RSTPP, which exhibits accurate mitochondrial-targeting ability, fast response, excellent selectivity and high sensitivity. Notably, using our probe the appearance of mitochondrial HOCl in macrophages during bacterial infection has been revealed for the first time, as confirmed with non-phagocytic cells and inhibitors as controls. Furthermore, the probe has a detection limit of down to 9 nM, which may enable it to monitor HOCl at trace levels and thus allow the study of the cellular function of mitochondrial HOCl under various bacterial infection events.
