# Supplementary material for: HOCl can appear in the mitochondria of macrophages during bacterial infection as revealed by a sensitive mitochondrial-targeting fluorescent probe
Source: Chem Sci. 2015 Jun 1;6(8):4884–8. doi: 10.1039/c5sc01562f (PMC5502397; doi:10.1039/c5sc01562f)
Supplement: Supplementary file 1 [file SC-006-C5SC01562F-s001.pdf]

Electronic Supplementary Material (ESI) for Chemical Science.
This journal is © The Royal Society of Chemistry 2015

Electronic Supplementary Information for

HOCl can appear in the mitochondria of macrophages during bacterial infection as revealed by a sensitive mitochondrial-targeting fluorescent probe

Jin Zhou, Lihong Li, Wen Shi,* Xinghui Gao, Xiaohua Li, and Huimin Ma*

1. Experimental

Reagents. 2-(4-Diethylamino-2-hydroxybenzoyl)benzoic acid, and 9-fluorenylmethyl chloroformate (Fmoc-Cl) were obtained from Adamas. 3-(1-Piperazzynyl) phenol was purchased from Alfa Aesar, and trifluoroacetic acid (TFA) from J & K. Thiourea, phosphorous oxychloride, triethylamine (TEA), *tert*-butyl hydroperoxide (TBHP), and piperidine were obtained from Beijing Chemical Company. Phosphate-buffered saline (PBS, pH 7.4), Dulbecco's Modified Eagle's medium (DMEM), fetal bovine serum (FBS), penicillin (100 U/mL), streptomycin (100 μg/mL), and LysoTracker Red DND-26 (DND-26) were obtained from Invitrogen Corporation. 3-(4,5-Dimethylthiazol-2-yl)-2,5-diphenyltetrazolium bromide (MTT) was obtained from Serva Electrophoresis GmbH. (4-Iodobutyl)triphenylphosphonium (IBTP) was prepared following the known procedure (Lin et al, *J. Biol. Chem.* 2002, 277, 17048–17056). Reduced glutathione (GSH), dimethyl sulphoxide (DMSO), L-cysteine, glucose, phorbol 12-myristate 13-acetate (PMA), lipopolysaccharide (LPS), rhodamine 123, and N-acetylcysteine (NAC) were purchased from Sigma-Aldrich. The preparation and/or concentration determination of *tert*-butoxy radical (TBO•) and other reactive oxygen species (HOCl, $ONOO^-$, NO, H_2O_2, •OH, NO_2^-, O_2^-, $^1O_2$, TBHP) were made according to the literatures (Li et al, *J. Am. Chem. Soc.* 2004, 126, 11543-11548; Chen et al, *Chem. Commun.* 2012, 48, 2949-2951; Liu et al, *Org. Lett.* 2013, 15, 878-881). The stock solution (1 mM) of RSTPP was prepared by dissolving an appropriate amount of RSTPP in DMSO. Ultrapure water (over 18 MΩ·cm) from a Milli-Q reference system (Millipore) was used throughout. All other solvents and reagents used were local products of anaylytica1 grade.

Apparatus. UV-vis absorption spectra were measured in 1×1 cm quartz cells with a TU-1900 spectrophotometer (Beijing, China). A Hitachi F-4600 spectrophotometer

(Tokyo, Japan) was used for fluorescence measurements with a 400 V PMT voltage. Fluorescence quantum yield (Φ_f) was determined with rhodamine 6G ($\Phi_f = 0.93$ in water) as a standard. ^{1}H NMR and ^{13}C NMR were measured on Bruker Avance-400 and Bruker Fourier-300 spectrometers. High resolution electrospray ionization mass spectra (HR-ESI-MS) were recorded on an APEX IV FTMS instrument (Bruker Daltonics), and matrix-assisted laser desorption ionization-time of flight (MALDI-TOF) mass spectra on a BIFLEX III instrument (Bruker, Bremen, Germany). The incubation was carried out in a shaker water bath (SKY-100C, Shanghai Sukun Industry & Commerce Company). The absorbance for MTT analysis was recorded on a microplate reader (BIO-TEK Synergy HT, USA) at 490 nm. Fluorescence imaging experiments were performed on FV 1000-IX81 (Olympus) confocal laser scanning microscope. Image processing was made with Olympus software (FV10-ASW) and ImageJ software (National Institutes of Health, USA).

Synthesis of RSTPP. As shown in Scheme 1, RSTPP was prepared by first synthesizing a rhodamine intermediate, then incorporating the S atom into the spirocyclic structure, and finally linking the mitochondrial-targeting moiety of triphenylphosphonium cation to the skeleton of rhodamine thiolactone.

In brief, 2-(4-diethylamino-2-hydroxybenzoyl)benzoic acid (**1**; 1.50 g, 4.8 mmol) and 3-(piperazin-1-yl)phenol (**2**; 853 mg, 4.8 mmol) were stirred in TFA (20 mL) for 3 h at 95 °C. After cooling to room temperature, the reaction mixture was poured into 300 mL of ether. The resulting red precipitate piperazine-rhodamine was collected and dried (Dickinson et al, *J. Am. Chem. Soc.* 2008, 130, 9638–9639; Guan et al, *Org. Biomol. Chem.* 2014, 12, 3944–3949). Then, the piperazine-rhodamine product (1.42 g), Fmoc-Cl (904 mg, 3.5 mmol), and $NaHCO_3$ (710 mg, 8.5 mmol) were added to a dry Schlenk flask containing 20 mL of dry acetonitrile under a nitrogen atmosphere, and stirred vigorously for 3 h at room temperature. The reaction mixture was then extracted with dichloromethane and washed with water for three times. The organic layer was dried over anhydrous Na_2SO_4, filtered and evaporated under reduced pressure, obtaining product **3**, which was used directly in next step.

Then, to a stirred solution of **3** (678 mg) in 1,2-dichloroethane (10 mL), phosphorus oxychloride (0.5 mL) was added dropwise. The mixture was refluxed for 4 h at 95 °C, followed by evaporation under reduced pressure. The residue as a violet-red oil was

dissolved in THF (6 mL), and the resulting solution was then added dropwise to a mixed solution of thiourea (304 mg, 4 mmol) and triethylamine (2.4 mL) in THF (10 mL)/water (2 mL). After stirring overnight at room temperature, the solvent was removed under reduced pressure to give a pink oil, followed by adding 10 mL of water. The formed precipitate was collected and dissolved in dichloromethane. The resulting dichloromethane solution was washed several times with water. After the solvent was removed under reduced pressure, the crude product was subjected to silica-gel column chromatography with petroleum ether (b.p. 60-90 ^{o}C)/ethyl acetate (6:1, v/v) as eluent, affording 152 mg of **4** as a white powder (yield 22%). The ^{1}H NMR and ^{13}C NMR spectra of **4** are shown below in Figures S1 and S2, respectively. ^{1}H NMR (300 MHz, $CDCl_3$): δ 7.88 (d, J = 6.6 Hz, 1H), 7.78 (d, J = 7.5 Hz, 2H), 7.61–7.30 (m, 8H), 7.20 (d, J = 7.5 Hz, 1H), 6.79 (d, J = 8.7 Hz, 1H), 6.72 (d, J = 8.4 Hz, 1H), 6.61–6.51 (m, 2H), 6.37–6.29 (m, 2H), 4.49 (d, J = 6.6 Hz, 2H), 4.27 (t, J = 6.5 Hz, 1H), 3.59 (s, 4H), 3.35 (q, J = 7.0 Hz, 4H), 3.15 (s, 4H), 1.18 (t, J = 7.0 Hz, 6H); ^{13}C NMR (75 MHz, $CDCl_3$): δ 197.4, 157.7, 155.1, 151.5, 148.6, 143.9, 141.4, 135.5, 134.4, 129.9, 127.8, 127.1, 124.9, 122.8, 120.1, 113.5, 112.4, 108.6, 108.1, 102.6, 97.5, 67.4, 62.0, 48.4, 47.4, 44.4, 43.5, 12.6. HR-ESI-MS (*m/z*): calcd for $C_{43}H_{39}N_3O_4S$, 694.2734 $[M+H]^+$; found 694.2738.

Finally, compound **4** (83.2 mg, 0.12 mmol) was dissolved in acetonitrile containing 15% piperidine, followed by stirring at room temperature for 30 min. The mixture was then evaporated under reduced pressure, and the resulting residue was mixed with IBTP (138 mg, 0.24 mmol), $NaHCO_3$ (120 mg, 0.96 mmol) and 15 mL of acetonitrile under a nitrogen atmosphere. The reaction solution was stirred for 24 h at room temperature, and then evaporated under reduced pressure. The residue was purified by silica-gel column chromatography with dichloromethane/methanol (1/10, v/v), affording RSTPP as a white solid (18 mg, yield 19%). The ^{1}H NMR and ^{13}C NMR spectra of RSTPP are shown below in Figures S3 and S4, respectively. ^{1}H NMR (400 MHz, $CDCl_3$): δ 7.98–7.75 (m, 10H), 7.71 (d, J = 5.6 Hz, 6H), 7.54 (t, J = 7.4 Hz, 1H), 7.47 (t, J = 7.4 Hz, 1H), 7.18 (d, J = 7.6 Hz, 1H), 6.73 (d, J = 8.8 Hz, 1H), 6.69 (d, J = 8.8 Hz, 1H), 6.55 (s, 1H), 6.50 (d, J = 9.2 Hz, 1H), 6.38–6.27 (m, 2H), 3.74 (t, J = 14.1 Hz, 2H), 3.33 (q, J = 6.8 Hz, 4H), 3.17 (s, 4H), 2.62 (s, 4H), 2.56 (t, J = 5.9 Hz, 2H), 2.02–1.91 (m, 2H), 1.74 (d, J = 7.2 Hz, 2H), 1.16 (t, J = 6.9 Hz, 6H). ^{13}C NMR (100 MHz, $CDCl_3$): δ 197.4, 157.6, 151.9, 151.5, 148.6, 135.2, 133.8, 133.7, 133.7, 130.5, 129.6, 127.3, 122.6, 117.7, 112.6, 111.7, 108.4, 108.1,

101.8, 97.6, 62.2, 56.5, 52.8, 47.9, 44.4, 12.6. HR-ESI-MS (*m/z*): calcd for $C_{50}H_{51}N_2O_3PS^+$, 788.3434 $[M]^+$; found 788.3431.

General Procedure for HOCl Detection. Unless otherwise stated, all the fluorescence measurements were made in PBS (pH 7.4) according to the following procedure. In a test tube, 4 mL of PBS and 50 μL of RSTPP stock solution (1 mM) were mixed, followed by addition of an appropriate volume of HOCl solution. The final volume was adjusted to 5 mL with PBS, and the reaction solution was mixed rapidly. After 2 min, a 3-mL portion of the reaction solution was transferred to a quartz cell of 1 cm optical length to measure the absorbance or fluorescence with $\lambda_{ex/em}$ = 553/580 nm. Results were expressed as mean ± standard deviation (n =3).

Cytotoxicity Assay. Cytotoxicity of RSTPP was tested on HeLa cells using a standard MTT assay, as described previously (Wan et al, *Angew. Chem. Int. Ed.* 2014, 53, 10916).

Cultivation of Cells and Bacteria. Raw264.7 cells (mouse leukemic monocyte macrophage) or HeLa cells were cultured in DMEM containing 10% FBS (fetal bovine serum) and antibiotics (100 units/mL penicillin and 100 μg/mL streptomycin) in a humidified atmosphere of 95% air and 5% CO_2 at 37 °C. *Escherichia coli* (*E. coli*) were cultured at 37 °C in Luria-Bertani (LB) culture media (pH 7.4), and agitated on a shaker water bath at a speed of 150 rpm for 8 h. Then, they were collected during the middle-exponential phase, washed three times by centrifuging for 10 min at 2000 rpm to separate from the nutrient with PBS and quantified by the absorbance at 600 nm.

Colocalization Fluorescence Imaging. In this experiment, cells (RAW264.7 or HeLa) were co-stained with RSTPP (10 μM) and Rhodamine 123 (500 nM) or DND-26 (500 nM) for 20 min at 37 °C. After washing with PBS for three times, the cells were exposed to 50 μM HOCl for 20 min. Before fluorescence imaging, the adherent cells were further washed three times with PBS (pH 7.4). Fluorescence imaging experiments were performed with excitations at 488 nm for both Rhodamine 123 and DND-26, and 559 nm for RSTPP, respectively; emissions were collected at 495-550 nm for both Rhodamine 123 and DND-26, and 570-670 nm for RSTPP, respectively.

Fluorescence Imaging of Endogenous HOCl in Living Cells. RAW264.7 cells were pretreated with LPS (1 μg/mL) for 12 h and then with PMA (1 μg/mL) for 1 h, followed by incubation with RSTPP (10 μM) for 20 min. Prior to imaging, the culture medium was

removed, and the treated cells were washed three times with PBS (pH 7.4), followed by fluorescence imaging with excitation at 559 nm and emission from 570 to 670 nm.

Fluorescence Imaging of Exogenous HOCl in Living Cells. HeLa cells was seeded in glass bottom dishes in culture medium with a density of 1×10^5 cells per dish, and incubated for 24 h. Then, the cells were incubated with 10 μM RSTPP for 20 min at 37 °C. After washing with PBS (pH 7.4) for three times, the cells were treated with HOCl (50 μM) for 10 min at 37 °C. Prior to imaging, the cells were washed three times with PBS (pH 7.4), followed by fluorescence imaging with excitation at 559 nm and emission from 570 to 670 nm.

Fluorescence Imaging of HOCl in Living Cells Infected by Bacteria. For bacterial infection of macrophages, *E. coli* at a concentration of about 5×10^6 CFU/mL was added to Raw264.7 cells, followed by culturing in an atmosphere of 95% air and 5% CO_2 at 37 °C for different periods of time. Then, the cells were washed three times with PBS (pH 7.4), and incubated with RSTPP (10 μM) for 20 min at 37 °C. Prior to fluorescence imaging, the cells were washed again with PBS for three times to remove superfluous RSTPP. The cells were imaged with excitation at 559 nm and emission from 570 to 670 nm.

2. Supporting Figures

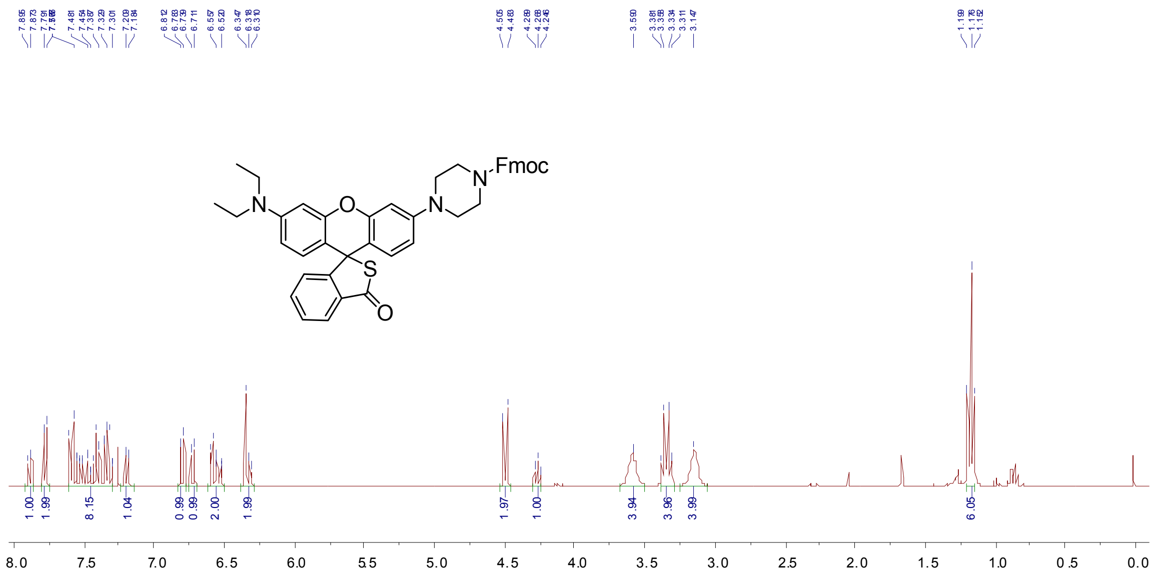

Figure S1. ^{1}H NMR spectrum of compound **4** (300 MHz, $CDCl_3$, 298 K).

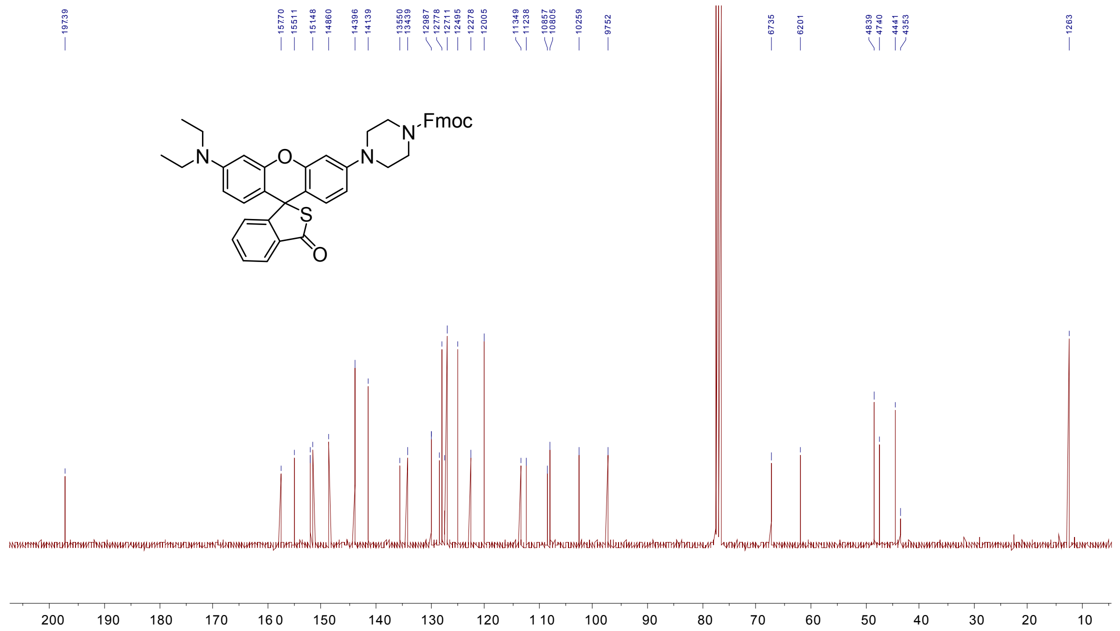

Figure S2. ^{13}C NMR spectrum of compound **4** (75 MHz, $CDCl_3$, 298 K).

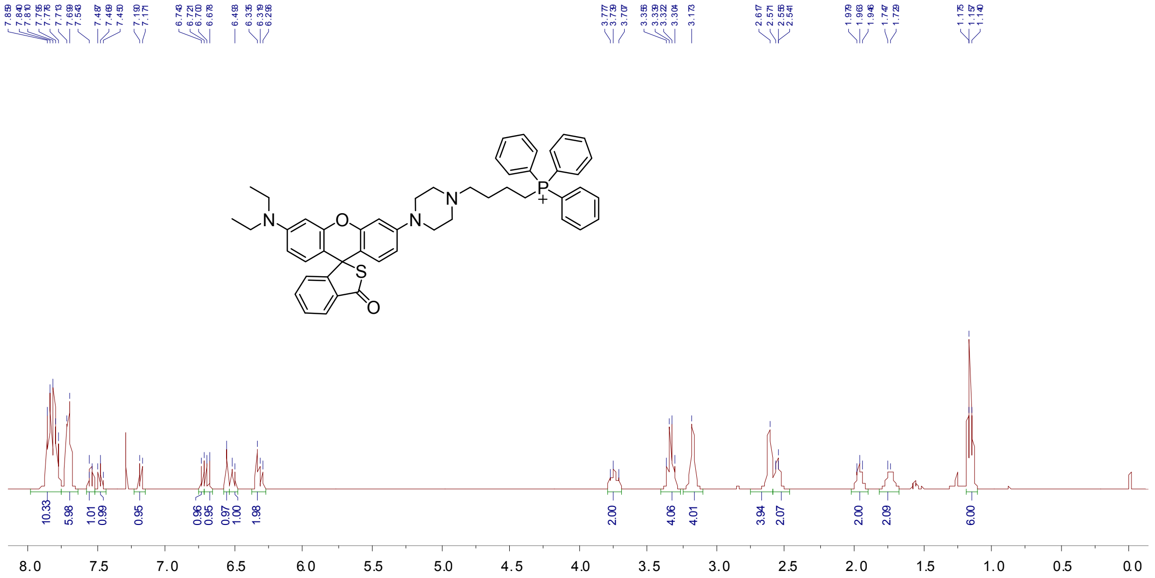

Figure S3. ^{1}H NMR spectrum of RSTPP (400 MHz, $CDCl_3$, 298 K).

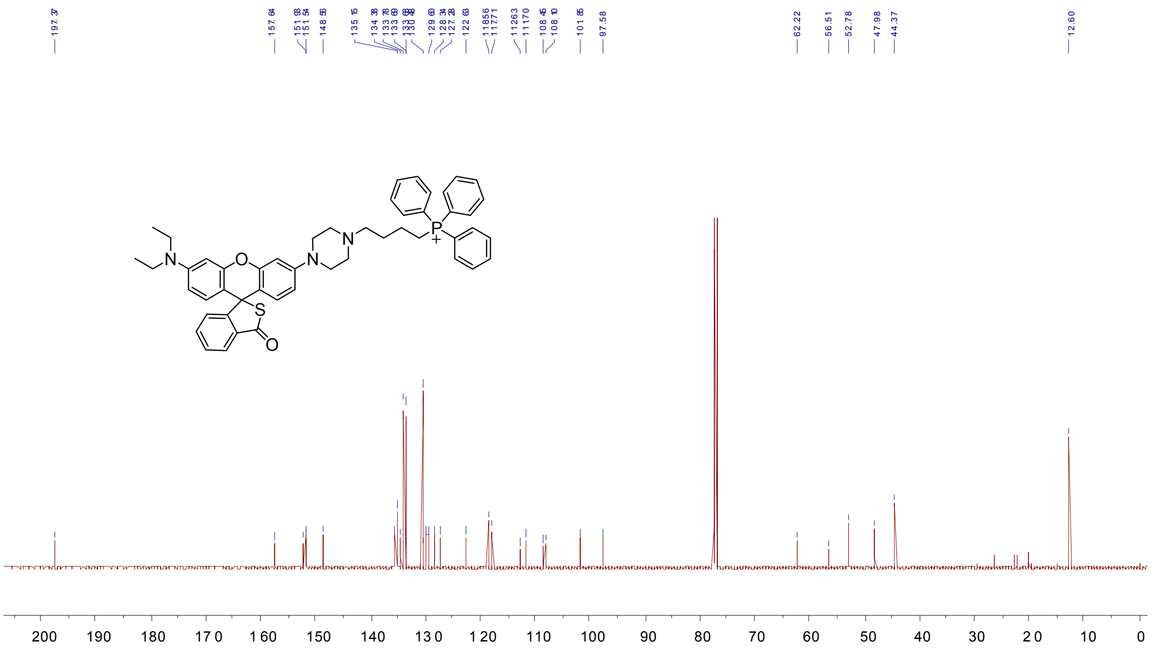

Figure S4. ^{13}C NMR spectrum of RSTPP (100 MHz, $CDCl_3$, 298 K).

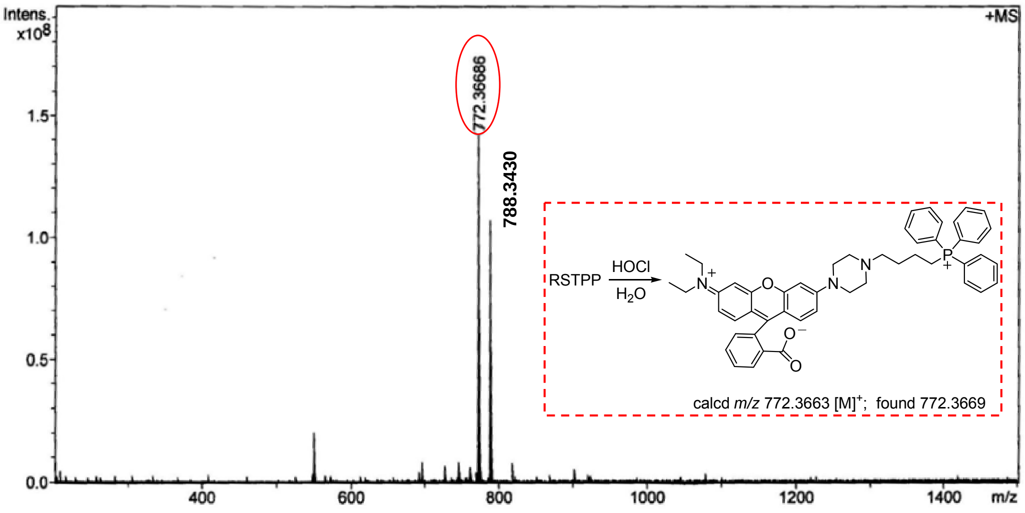

Figure S5. MALDI-TOF mass spectrum of RSTPP (10 μM) reacting with HOCl (10 μM) for 5 min. The peak at m/z = 788.3430 is the remaining RSTPP.

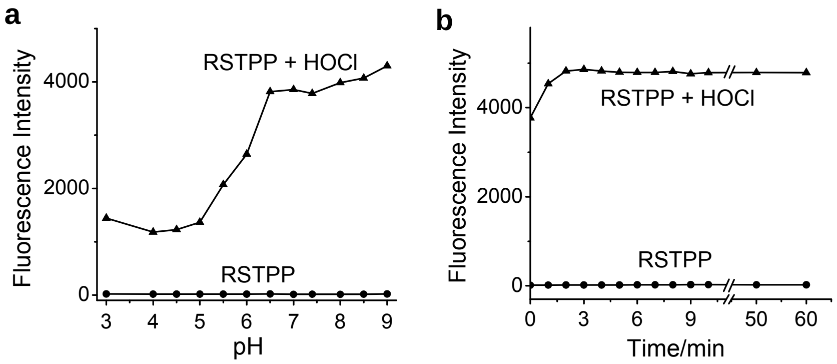

Figure S6. Effects of pH (**a**) and time (**b**) on the fluorescence of RSTPP (10 μM) with (triangle) and without (circle) HOCl (100 μM). The fluorescence (panel b) was measured at pH 7.4. $\lambda_{ex/em}$ = 553/580 nm.

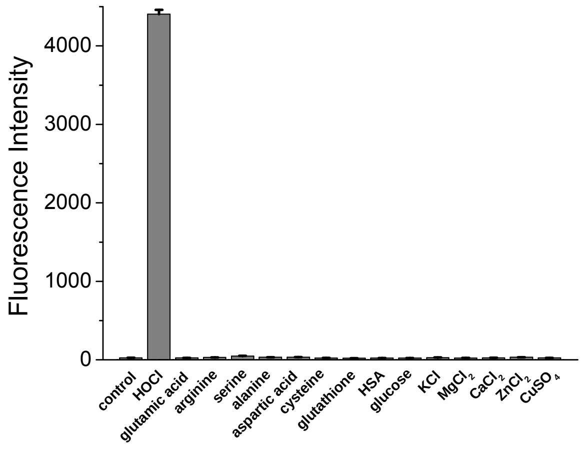

Figure S7. Fluorescence responses of RSTPP (10 μM) to other biologically relevant species in PBS (pH 7.4): HOCl (100 μM), glutamic acid (1 mM), arginine (1 mM), serine (1 mM), alanine (1 mM), aspartic acid (1 mM), cysteine (5 mM), glutathione (5 mM), HSA (human serum albumin, 100 μM), glucose (10 mM), KCl (150 mM), $MgCl_2$ (2 mM), $CaCl_2$ (2 mM), $ZnCl_2$ (100 μM), $CuSO_4$ (100 μM). $\lambda_{ex/em}$ = 553/580 nm.

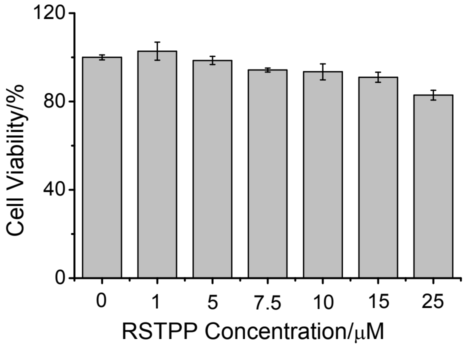

Figure S8. Effects of RSTPP at varied concentrations (0–25 μM) on the viability of HeLa cells. The viability of cells without RSTPP is defined as 100%. The results are the mean ± standard deviation of five separate measurements. As is seen, less than 15 μM of RSTPP hardly affects the cell viability, showing low cytotoxicity and good biocompatibility.

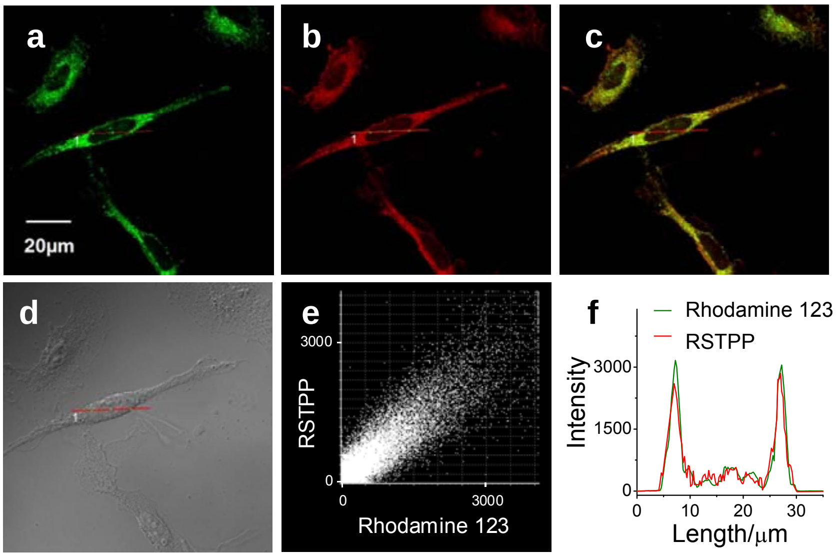

Figure S9. Colocalization of Rhodamine 123 and RSTPP in HeLa cells. Cells were co-stained with Rhodamine 123 (500 nM) and RSTPP (10 μM) at 37 °C for 20 min, and then treated with HOCl (50 μM) for 10 min. (**a**) Fluorescence image from Rhodamine 123 channel (λ_{ex} = 488 nm, λ_{em} = 495–550 nm). (**b**) Fluorescence image from RSTPP channel (λ_{ex} = 559 nm, λ_{em} = 570–670 nm). (c) Merged image of images (**a**) and (**b**). (**d**) Corresponding differential interference contrast (DIC) image. (**e**) Intensity correlation plot of Rhodamine 123 and RSTPP (a high Pearson's coefficient of 0.91 and an overlap coefficient of 0.93 are obtained from this plot). (**f**) Intensity profiles of Rhodamine 123 and RSTPP within the linear ROI 1 (red lines in **Figure S9a** and **Figure S9b**) across the HeLa cell.

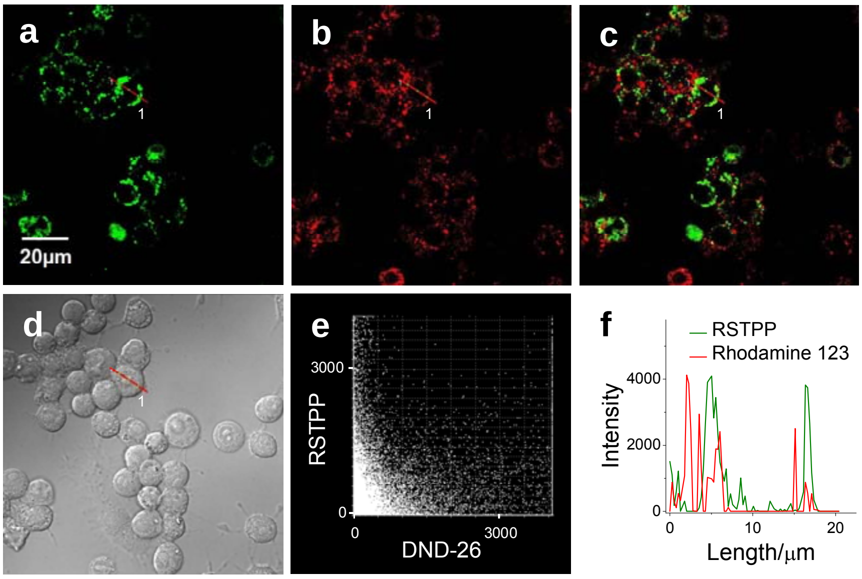

Figure S10. Colocalization of DND-26 and RSTPP in RAW264.7 cells. Cells were co-stained with DND-26 (500 nM) and RSTPP (10 μM) at 37 °C for 20 min, and then treated with HOCl (50 μM) for 10 min. (**a**) Fluorescence image from DND-26 channel (λ_{ex} = 488 nm, λ_{em} = 495–550 nm). (**b**) Fluorescence image from RSTPP channel (λ_{ex} = 559 nm, λ_{em} = 570–670 nm). (**c**) Merged image of images (**a**) and (**b**). (**d**) Corresponding DIC image. (**e**) Intensity correlation plot of DND-26 and RSTPP (a rather poor Pearson's coefficient of 0.30 and an overlap coefficient of 0.28 are obtained from this plot). (**f**) Intensity profiles of DND-26 and RSTPP within the linear ROI 1 (red lines in **Figure S10a** and **Figure S10b**) cross the RAW264.7 cell.

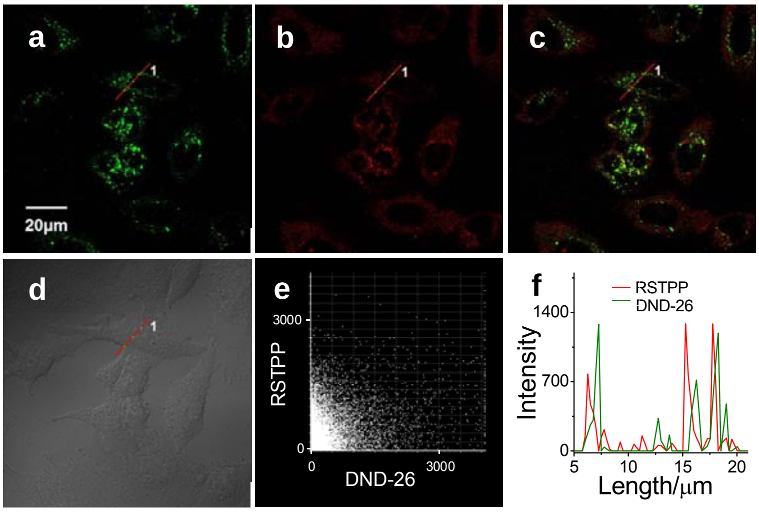

Figure S11. Colocalization of DND-26 and RSTPP in HeLa cells. HeLa cells were co-stained with DND-26 (500 nM) and RSTPP (10 μM) at 37 °C for 20 min, and

then treated with HOCl (50 μM) for 10 min. (**a**) Fluorescence image from DND-26 channel (λ_{ex} = 488 nm, λ_{em} = 495–550 nm). (**b**) Fluorescence image from RSTPP channel (λ_{ex} = 559 nm, λ_{em} = 570–670 nm). (**c**) Merged image of images (**a**) and (**b**). (**d**) Corresponding DIC image. (**e**) Intensity correlation plot of DND-26 and RSTPP (a rather poor Pearson's coefficient of 0.24 and an overlap coefficient of 0.27 are obtained from this plot). (**f**) Intensity profiles of DND-26 and RSTPP within the linear ROI 1 (red lines in **Figure S11a** and **Figure S11b**) cross the HeLa cell.

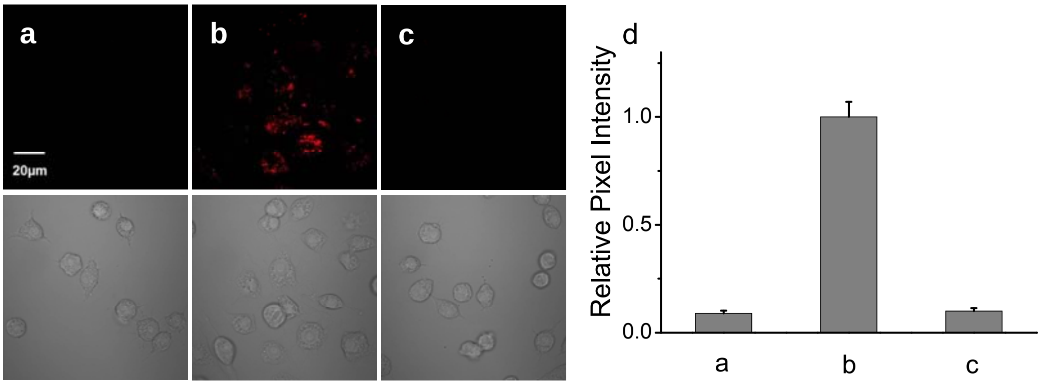

Figure S12. Confocal fluorescence images of RAW264.7 cells under different conditions. (**a**) The cells were stained with 10 μM RSTPP for 20 min at 37 °C (control). (**b**) The cells were pretreated with LPS (1 μg/mL) for 12 h, then with PMA (1 μg/mL) for 1 h, and finally stained with 10 μM RSTPP for 20 min at 37 °C. (**c**) The cells pretreated with LPS (1 μg/mL) and PMA (1 μg/mL) were incubated with 10 μM NAC for 10 min, and then with 10 μM RSTPP for 20 min at 37 °C. The DIC images of the corresponding samples are shown at the bottom; scale bar, 20 μm. (**d**) Relative pixel intensities of fluorescence images **a-c** (the pixel intensity from the image **b** is defined as 1.0). The results are the mean ± standard deviation of three separate measurements.

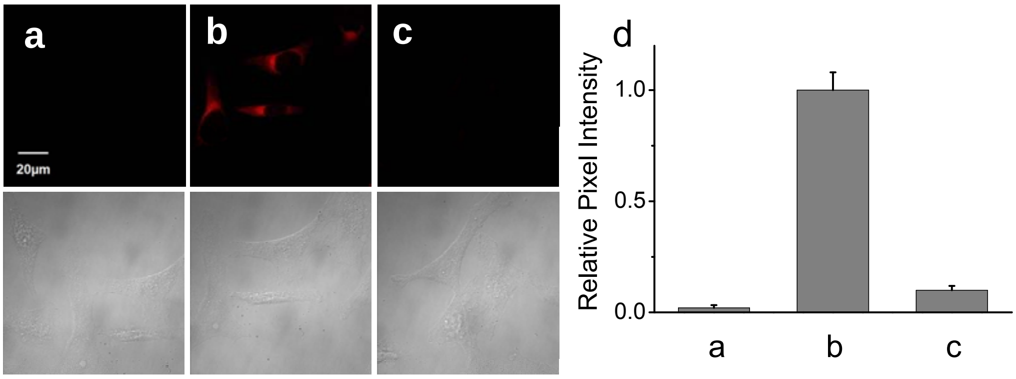

Figure S13. Confocal fluorescence images of HeLa cells under different conditions. (**a**) The cells were stained with 10 μM RSTPP for 20 min at 37 °C (control). (**b**) The RSTPP-loaded cells were incubated with 50 μM HOCl for 10 min. (**c**) The RSTPP-loaded cells were pretreated with 10 μM NAC for 10 min, and then incubated with 50 μM HOCl for 10 min at 37 °C. The DIC images of the corresponding samples are shown at the bottom; scale bar, 20 μm. (**d**) Relative pixel intensities of fluorescence images **a-c** (the pixel intensity from the image **b** is defined as 1.0). The results are the mean ± standard deviation of three separate measurements. As shown in **Figure S13a**, without HOCl the probe-loaded cells show negligible intracellular background fluorescence, whereas strong fluorescence is observed after treatment with HOCl (**Figure S13b**), which suggests that HOCl can enter the mitochondria and react with the probe producing fluorescence. Notably, the RSTPP-loaded cells treated with NAC and then with HOCl show a largely decreased fluorescence (**Figure S13c**), indicating that the fluorescence of the probe-loaded cells indeed arises from the action of HOCl.

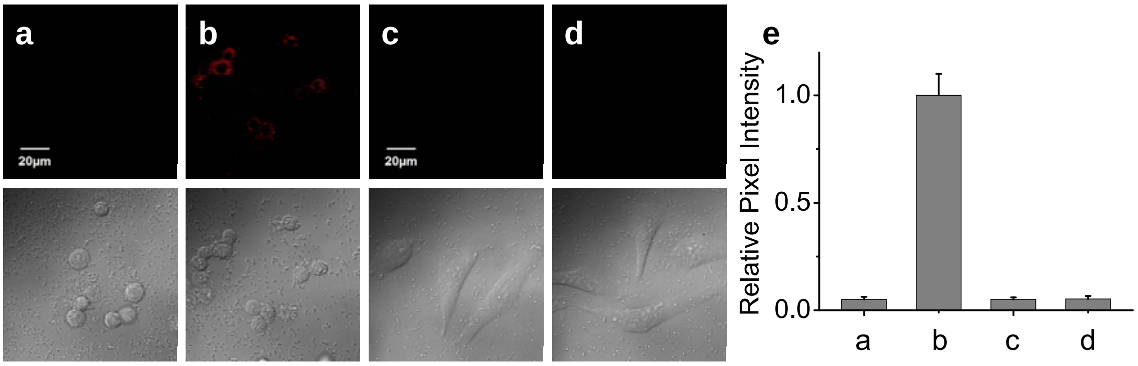

Figure S14. Confocal fluorescence images of (images **a** and **b**) RAW264.7 and (images **c** and **d**) HeLa cells during *E. coli* infection. (**a**) RAW264.7 cells were

treated with *E. coli* at a concentration of 5×10^6 CFU/mL for 7 h (control). (**b**) RAW264.7 cells were treated with *E. coli* at a concentration of 5×10^6 CFU/mL for 7 h, and then incubated with 10 μM RSTPP for 20 min. (**c**) HeLa cells were treated with *E. coli* at a concentration of 5×10^6 CFU/mL for 7 h (control). (**d**) HeLa cells were treated with *E. coli* at a concentration of 5×10^6 CFU/mL for 7 h, and then incubated with 10 μM RSTPP for 20 min. The DIC images of the corresponding samples are shown at the bottom; scale bar, 20 μm. (**e**) Relative pixel intensity measurements (n = 3) from images **a–d** by the software ImageJ. The pixel intensity from image **b** is defined as 1.0. As is seen, RAW264.7 cells infected by *E. coli* show strong fluorescence in the mitochondria, whereas infected HeLa cells do not.
